# Supplementary material for: Beyond Binary: (Re)Defining “Gender” for 21st Century Disaster Risk Reduction Research, Policy, and Practice
Source: Int J Environ Res Public Health. 2019 Oct 18;16(20):3984. doi: 10.3390/ijerph16203984 (PMC6843806; doi:10.3390/ijerph16203984)
Supplement: Supplementary file 1 [file ijerph-16-03984-s001.pdf]

Supplementary Material:

File S1. Included papers (*n* = 260).

Asterix (*) denotes journal article that appear to utilise sex and gender terms interchangeably.

First Author Surname (A-Z)	YEAR	Title of Journal Article	More than 2 Genders	Sex/ Sexes	Female	Male	Woman/Women	Man/ Men	Wife	Husband	Gay/Homosexual
**Adams***	2015	Comorbidity of PTSD, Major Depression, and Substance Use Disorder Among Adolescent Victims of the Spring 2011 Tornadoes in Alabama and Joplin, Missouri			**YES**	**YES**					
**Adeagbo***	2016	Effects of natural disasters on social and economic well being: A study in Nigeria			**YES**	**YES**	**YES**	**YES**			
**Aitken***	2015	Emergency Department Presentations following Tropical Cyclone Yasi			**YES**	**YES**					
**Akerkar**	2017	Gender, place and mental health recovery in disasters: Addressing issues of equality and difference					**YES**	**YES**			
**Alshehri**	2016	Public perceptions and attitudes to biological risks: Saudi Arabia and regional perspectives					**YES**	**YES**			
**Andersen***	2016	Gender and Climate Change in Latin America: An Analysis of Vulnerability, Adaptation and Resilience Based on Household Surveys			**YES**	**YES**	**YES**	**YES**			
**Ardagh***	2015	A sex disparity among Earthquake Victims		**YES**	**YES**	**YES**	**YES**	**YES**			
**Armas***	2017	Self-efficacy, stress, and locus of control: The psychology of earthquake risk perception in Bucharest, Romania		**YES**			**YES**	**YES**			

**Arnberg**	2015	Posttraumatic stress in survivors 1 month to 19 years after an airliner emergency landing		**YES**	**YES**	**YES**					
**Asif Malik**	2018	Prevalence of depression in earthquake 2005 affected areas of Muzaffarabad City (Azad Kashmir)		**YES**	**YES**	**YES**					
**Ahsan***	2016	Burr Hole Evacuation of Extradural Hematoma in Mass Trauma. A Life Saving and Time Saving Procedure: Our Experience in the Earthquake of 2005			**YES**	**YES**					
**Austin**	2016	Disaster devastation in poor nations: The direct and indirect effects of gender equality, ecological losses, and development					**YES**	**YES**			
**Abdurrahman**	2015	Neurosurgical Injuries Caused by the 2011 Van Earthquake: The Experience at the Van Regional Training and Research Hospital		**YES**	**YES**	**YES**					
**Ayub***	2015	Rate and predictors of psychotic symptoms after Kashmir earthquake			**YES**	**YES**	**YES**	**YES**			
**Bala***	2017	Sentiment trends on natural disasters using location based twitter opinion mining			**YES**	**YES**					
**Ballera**	2015	Management of the dead in Tacloban City after Typhoon Haiyan		**YES**	**YES**	**YES**					
**Ben Khelil***	2017	A comparison of suicidal behavior by burns five years before and five years after the 2011 Tunisian Revolution		**YES**	**YES**	**YES**					
**Bhandari***	2017	Psychological distress among the adult survivors of Kathmandu valley from Nepal's 2015 earthquake			**YES**	**YES**					
**Bhattarai***	2018	Determinants of resilience among people who sustained			**YES**	**YES**					

		spinal cord injury from the 2015 earthquake in Nepal									
**Bianchini***	2017	Moderate Depression Promotes Posttraumatic Growth (Ptg): A Young Population Survey 2 Years after the 2009 L'Aquila Earthquake			**YES**	**YES**					
**Bolt***	2018	The Associations between Self-Reported Exposure to the Chernobyl Nuclear Disaster Zone and Mental Health Disorders in Ukraine		**YES**	**YES**	**YES**	**YES**	**YES**			
**Bradshaw**	2015	Gendered Rights in the Post-2015 Development and Disasters Agendas					**YES**	**YES**			
**Cadichon***	2017	Symptoms of PTSD Among Adolescents and Young Adult Survivors Six Years after the 2010 Haiti Earthquake		**YES**	**YES**	**YES**	**YES**	**YES**			
**Gaillard**	2017	Beyond men and women: a critical perspective on gender and disaster	**YES**				**YES**	**YES**			
**Bradshaw**	2015	Engendering development and disasters					**YES**	**YES**			
**Moreno**	2016	Disaster Resilience: Addressing Gender Disparities					**YES**	**YES**			
**Chen***	2016	A 10-year follow-up study on suicidal mortality after 1999 Taiwan earthquake			**YES**	**YES**					
**Felix***	2019	Addressing Disaster Exposure Measurement Issues With Latent Class Analysis			**YES**	**YES**					
**Chiang***	2017	Adjunctive hyperbaric oxygen therapy in severe burns: Experience in Taiwan Formosa Water Park dust explosion disaster		**YES**	**YES**	**YES**	**YES**	**YES**			
**Danielson***	2017	Adolescent Substance Use Following a Deadly U.S. Tornado Outbreak: A			**YES**	**YES**	**YES**	**YES**			

		Population-Based Study of 2,000 Families									
**Campbell***	2016	Violence and abuse of internally displaced women survivors of the 2010 Haiti earthquake			**YES**	**YES**	**YES**	**YES**			
**Cao***	2016	DSM-5 Posttraumatic Stress Disorder Symptom Structure in Disaster-Exposed Adolescents: Stability across Gender and Relation to Behavioral Problems			**YES**	**YES**					
**Cao***	2017	Real-time Acute Stress Facilitates Allocentric Spatial Processing in a Virtual Fire Disaster			**YES**	**YES**					
**Carmassi***	2015	Impact of DSM-5 PTSD and gender on impaired eating behaviors in 512 Italian earthquake survivors		**YES**	**YES**	**YES**	**YES**	**YES**			
**Casey**	2015	Evaluations of reproductive health programs in humanitarian settings: a systematic review					**YES**	**YES**			
**Chang***	2017	Comorbid diseases as risk factors for incident posttraumatic stress disorder (PTSD) in a large community cohort (KCIS no.PSY4)		**YES**	**YES**	**YES**	**YES**	**YES**			
**Chaudhary***	2017	Humanitarian response to reproductive and sexual health needs in a disaster: the Nepal Earthquake 2015 case study			**YES**	**YES**	**YES**	**YES**			
**Chen***	2018	A Correlational Study of Acute Stress and Resilience Among Hospitalized Burn Victims Following the Taiwan Formosa Fun Coast Explosion			**YES**	**YES**					
**Chui***	2017	Predictive factors of depression symptoms among adolescents in the 18-month follow-up after Wenchuan earthquake in China		**YES**	**YES**	**YES**					
**Chung***	2017	Gender-sensitive disaster vulnerability using analytic			**YES**	**YES**	**YES**	**YES**			

		hierarchy process and genetic algorithm									
**Chunpeng***	2017	Prenatal Earthquake Exposure and Midlife Uric Acid Levels Among Chinese Adults		**YES**		**YES**	**YES**	**YES**			
**Ciocca***	2015	Post-traumatic stress disorder, coping strategies and type 2 diabetes: psychometric assessment after L'Aquila earthquake			**YES**	**YES**	**YES**	**YES**			
**Codreanu***	2015	Factors Associated with Discussion of Disasters by Final Year High School Students: An International Cross-sectional Survey		**YES**	**YES**	**YES**					
**Cohen**	2017	Comparison of Simulated Treatment and Cost-effectiveness of a Stepped Care Case-Finding Intervention vs Usual Care for Posttraumatic Stress Disorder After a Natural Disaster		**YES**	**YES**	**YES**					
**Cui***	2018	Resilience of an Earthquake-Stricken Rural Community in Southwest China: Correlation with Disaster Risk Reduction Efforts			**YES**	**YES**					
**Cvetkovic***	2018	The Role of Gender in Preparedness and Response Behaviors towards Flood Risk in Serbia		**YES**	**YES**	**YES**	**YES**	**YES**			
**Dahal***	2018	Prevalence and risk factors of post-traumatic stress disorders among the survivors of 2015 Nepal earthquake, in Dhading, Nepal			**YES**	**YES**					
**Dai***	2016	The incidence of post-traumatic stress disorder among survivors after earthquakes:a systematic review and meta-analysis			**YES**	**YES**					

**Dominey-Howes, D., Gorman-Murray, A., & McKinnon, S.**	2018	On the disaster experiences of sexualand gender (LGBTI) minorities: insights to support inclusive disaster risk reduction policy and practice	**YES**	**YES**	**YES**	**YES**		**YES**			**YES**
**Dorahy***	2015	Impact of average household income and damage exposure on post-earthquake distress and functioning: A community study following the February 2011 Christchurch earthquake			**YES**	**YES**					
**Doyle***	2018	Motivations to prepare after the 2013 Cook Strait Earthquake, N.Z			**YES**	**YES**					
**Du Na***	2019	Post-traumatic stress disorder and depression symptoms of adolescents survived from a seriously-hit area in China: A 3-year follow-up study			**YES**	**YES**					
**Eiser***	2015	Risk perceptions and trust following the 2010 and 2011 Icelandic volcanic ash crises			**YES**	**YES**					
**Eisma***	2019	Complicated grief and post-traumatic stress symptom profiles in bereaved earthquake survivors: a latent class analysis			**YES**	**YES**	**YES**	**YES**			
**Ejeta**	2018	Community's Emergency Preparedness for Flood Hazards in Dire-dawa Town, Ethiopia: A Qualitative Study			**YES**	**YES**					
**Elliot***	2017	Beyond disasters: A longitudinal analysis of natural hazards' unequal impacts on residential instability			**YES**	**YES**	**YES**	**YES**			
**Fan***	2017	Association of Val66Met polymorphism at brain derived neurotrophic factor gene with depression among Chinese adolescents after Wenchuan earthquake: An 18months longitudinal study			**YES**	**YES**					

**Ferhusson***	2015	Perceptions of distress and positive consequences following exposure to a major disaster amongst a well-studied cohort			**YES**	**YES**	**YES**	**YES**			
**Fernandez***	2018	Factors influencing fire, earthquake, and cyclone risk perception in Yangon, Myanmar			**YES**	**YES**					
**Forbes***	2015	The role of anger and ongoing stressors in mental health following a natural disaster			**YES**	**YES**	**YES**	**YES**			
**Fox***	2017	Hazard Experience, Geophysical Vulnerability, and Flood Risk Perceptions in a Postdisaster City, the Case of New Orleans			**YES**	**YES**	**YES**	**YES**			
**Frankenberg***	2017	Adult Mortality Five Years after a Natural Disaster		**YES**	**YES**	**YES**	**YES**	**YES**			
**Fukushi***	2018	Mental and physical stress of the Fukushima disaster evacuees as estimated by the measurement of urinary 8-hydroxy-2'-deoxyguanosine			**YES**	**YES**					
**Gaillard**	2017	Sexual and gender minorities in disaster	**YES**				**YES**	**YES**			
**Gallagher***	2017	Dyadic effects of attachment on mental health: Couples in a postdisaster context			**YES**	**YES**					
**Gao***	2015	Health-related quality of life of fracture victims four years after the 2008 Sichuan earthquake			**YES**	**YES**					
**Garcia**	2016	Coping, rumination and posttraumatic growth in people affected by an earthquake					**YES**	**YES**			
**Gorman-Murray**	2018	Listening and learning: giving voice to trans experiences of disasters	**YES**				**YES**	**YES**			**YES**
**Goryoda**	2018	Social Capital and Dietary Intakes Following the 2011 Great East Japan Earthquake and Tsunami					**YES**	**YES**			
**Greaves**	2015	Regional differences in the psychological recovery of					**YES**	**YES**			

		Christchurch residents following the 2010/2011 earthquakes: a longitudinal study									
**Groves***	2017	Descriptive study of earthquake-related spinal cord injury in Nepal			**YES**	**YES**	**YES**	**YES**			
**Guo***	2017	Post-traumatic stress disorder and depression among adult survivors 8 years after the 2008 Wenchuan earthquake in China			**YES**	**YES**					
**Hall**	2019	The association between disaster exposure and media use on post-traumatic stress disorder following Typhoon Hato in Macao, China					**YES**	**YES**			
**Hamama-Raz***	2015	Gender differences in psychological reactions to Hurricane Sandy among New York Metropolitan Area residents			**YES**	**YES**	**YES**	**YES**			
**Han***	2019	Social support moderates association between posttraumatic growth and trauma-related psychopathologies among victims of the Sewol Ferry Disaster			**YES**	**YES**	**YES**	**YES**			
**Hanaoka**	2018	Do risk preferences change? Evidence from the Great East Japan Earthquake					**YES**	**YES**			
**Hancock**	2016	Positive post-disaster images: A daydream machine?					**YES**	**YES**			
**Hashimoto**	2017	Influence of post-disaster evacuation on incidence of metabolic syndrome					**YES**	**YES**			
**Hayashi**	2017	The impact of evacuation on the incidence of chronic kidney disease after the Great East Japan Earthquake: The Fukushima Health Management Survey					**YES**	**YES**			

**Heetkamp***	2015	PTSD and resilience in adolescents after New Zealand earthquakes			**YES**	**YES**	**YES**	**YES**			
**Heid***	2016	Vulnerable, But Why? Post-Traumatic Stress Symptoms in Older Adults Exposed to Hurricane Sandy			**YES**	**YES**	**YES**	**YES**			
**Helander***	2016	Analysis of disaster risk attitudes in situation awareness: A cultural and gender perspective			**YES**	**YES**	**YES**	**YES**			
**Hirani**	2018	Breastfeeding in Disaster Relief Camps: An Integrative Review of Literature									
**Horikoshi**	2016	Residence-related factors and psychological distress among evacuees after the Fukushima Daiichi nuclear power plant accident: a cross-sectional study					**YES**	**YES**			
**Hu***	2015	Recovery from post-traumatic stress disorder after a flood in China: a 13-year follow-up and its prediction by degree of collective action			**YES**	**YES**					
**Hu***	2018	Assessment on the burden of bacillary dysentery associated with floods during 2005-2009 in Zhengzhou City, China, using a time-series analysis			**YES**	**YES**					
**Huang***	2017	A preliminary report on psychiatric impairments and quality of life among Kaohsiung gas explosion victims 6 months after the event			**YES**	**YES**	**YES**	**YES**			
**Hudson***	2019	An evaluation and monetary assessment of the impact of flooding on subjective well-being across genders in Vietnam			**YES**	**YES**	**YES**	**YES**			
**Hugelius***	2017	Facebook Enables Disaster Research Studies: The Use of Social Media to Recruit			**YES**	**YES**	**YES**	**YES**			

		Participants in a Post-Disaster Setting									
**Ibrahim***	2019	Vulnerability to recurrent shocks and disparities in gendered livelihood diversification in remote areas of Nigeria			**YES**	**YES**	**YES**	**YES**			
**Igarashi***	2018	Long-term outcomes of patients evacuated from hospitals near the Fukushima Daiichi nuclear power plant after the Great East Japan Earthquake		**YES**	**YES**	**YES**	**YES**	**YES**			
**Illiyas***	2018	Hazard-centric evacuation experiments - A study on occupant responses to earthquake alarm			**YES**	**YES**					
**Inatomi**	2017	Clinical characteristics of patients seizure following the 2016 Kumamoto earthquake		**YES**	**YES**	**YES**					
**Inoue***	2018	Gender-Based Risk and Protective Factors for Psychological Distress in the Midterm Recovery Period Following the Great East Japan Earthquake		**YES**	**YES**	**YES**	**YES**	**YES**			
**Ishikura***	2016	Public Attitudes toward an Epidemiological Study with Genomic Analysis in the Great East Japan Earthquake Disaster Area		**YES**	**YES**	**YES**	**YES**	**YES**			
**Isik**	2015	Are women in Turkey both risks and resources in disaster management?	**YES**				**YES**	**YES**			
**Jamali**	2018	Post-disaster place attachment: A qualitative study of place attachment in the wake of the 2013 Moore tornado					**YES**	**YES**			
**Jang***	2017	Increased beta power in the bereaved families of the Sewol ferry disaster: A paradoxical compensatory phenomenon? A		**YES**	**YES**	**YES**	**YES**	**YES**			

		two-channel electroencephalography study									
**Jeon***	2017	Natural Course of Posttraumatic Symptoms in Late-Adolescent Maritime Disaster Survivors: Results of A 12-Month Follow-Up Study			**YES**	**YES**					
**Jia***	2017	Longitudinal Relationships between Social Support and Posttraumatic Growth among Adolescent Survivors of the Wenchuan Earthquake			**YES**	**YES**					
**Jin**	2018	The relationship between PTSD, depression and negative life events: Ya'an earthquake three years later									
**Jonsson***	2017	The state of the residential fire fatality problem in Sweden: Epidemiology, risk factors, and event typologies			**YES**	**YES**					
**Kalina***	2016	A comparison of burn related injuries following the natural disaster Super Storm Hurricane Sandy to the National Burn Repository of the American Burn Association			**YES**	**YES**					
**Kamara***	2018	Resilience to Climate-Induced Disasters and Its Overall Relationship to Well-Being in Southern Africa: A Mixed-Methods Systematic Review			**YES**	**YES**	**YES**	**YES**			
**Kanakis***	2016	Preparing for disaster: Preparedness in a flood and cyclone prone community			**YES**	**YES**					
**Kane***	2017	Mental health and psychosocial problems in the aftermath of the Nepal earthquakes: findings from a representative cluster sample survey			**YES**	**YES**					
**Kiani**	2017	Personal factors affecting ethical performance in healthcare					**YES**	**YES**			

		workers during disasters and mass casualty incidents in Iran: a qualitative study									
**Kiani***	2016	The relationship between timing of admission to a hospital and severity of injuries following 2005 Pakistan earthquake		**YES**	**YES**	**YES**	**YES**	**YES**			
**Kpanake***	2018	Haitian people's expectations regarding post-disaster humanitarian aid teams' actions			**YES**	**YES**	**YES**	**YES**			
**Krishnan***	2019	Water, sanitation and hygiene (WASH) and disaster recovery for community resilience: A mixed methods study from Odisha, India			**YES**	**YES**	**YES**	**YES**			
**Kulkarni***	2017	Evacuations as a Result of Hurricane Sandy: Analysis of the 2014 New Jersey Behavioral Risk Factor Survey			**YES**	**YES**	**YES**	**YES**			
**Kuroda***	2017	Occurrence of depressive tendency and associated social factors among elderly persons forced by the Great East Japan Earthquake and nuclear disaster to live as long-term evacuees: a prospective cohort study			**YES**	**YES**	**YES**	**YES**			
**Labra***	2019	Men's Help-Seeking Attitudes in Rural Communities Affected by a Natural Disaster			**YES**	**YES**	**YES**	**YES**			
**Lebowitz**	2016	Cross-Sectional Data Within 1 Year of the Fukushima Meltdown: Effect-Size of Predictors for Depression		**YES**	**YES**	**YES**					
**Lee***	2017	Factors associated with post-traumatic stress symptoms among adolescents exposed to the Sewol ferry disaster in Korea			**YES**	**YES**					
**Lee***	2018	Factors Associated with Post-traumatic Stress Symptoms in Students Who Survived 20			**YES**	**YES**					

		Months after the Sewol Ferry Disaster in Korea									
**Lee***	2017	Social Support as a Mediator of Posttraumatic Embitterment and Perceptions of Meaning in Life among Danwon Survivors of the Sewol Ferry Disaster		**YES**	**YES**	**YES**					
**Lewis**	2016	Sustainable development through a gendered lens: climate change adaptation and disaster risk reduction					**YES**	**YES**			
**Li-Tsang***	2015	Rehabilitation needs of the survivors of the 2013 Ya'an earthquake in China			**YES**	**YES**					
**Li***	2015	Long-term effects of earthquake experience of young persons on cardiovascular disease risk factors			**YES**	**YES**					
**Liddell***	2018	Predictors of Individual Resilience Characteristics Among Individuals Ages 65 and Older in Post-Disaster Settings			**YES**	**YES**					
**Lin***	2015	Serological investigation to identify risk factors for post-flood infectious diseases: a longitudinal survey among people displaced by Typhoon Morakot in Taiwan			**YES**	**YES**					
**Liu***	2015	Analysis of Risk and Burden of Dysentery Associated with Floods from 2004 to 2010 in Nanning, China			**YES**	**YES**					
**Logie***	2016	My body is mine': Qualitatively exploring agency among internally displaced women participants in a small-group intervention in Leogane, Haiti			**YES**	**YES**	**YES**	**YES**		**YES**	
**Lowe***	2016	Mental Health Service Need and Use in the Aftermath of Hurricane Sandy: Findings in a			**YES**	**YES**	**YES**	**YES**			

		Population-Based Sample of New York City Residents									
**Luo***	2016	A study of farmers' flood perceptions based on the entropy method: an application from Jianghan Plain, China			**YES**	**YES**	**YES**	**YES**			
**Maheen**	2017	Rural Women's Experience of Living and Giving Birth in Relief Camps in Pakistan					**YES**	**YES**			
**Martin-Simpson**	2018	Measuring the benefits of using market based approaches to provide water and sanitation in humanitarian contexts					**YES**	**YES**			
**Matsumoto**	2015	Implications for Social support on prolonged sleep difficulties among a disaster-affected population: Second report from a cross-sectional survey in Ishinomaki, Japan		**YES**	**YES**	**YES**					
**McKinnon**	2017	Disasters, Queer Narratives, and the News: How Are LGBTI Disaster Experiences Reported by the Mainstream and LGBTI Media?	**YES**				**YES**	**YES**			
**McKinnon**	2017	Remembering an epidemic during a disaster: memories of HIV/AIDS, gay male identities and the experience of recent disasters in Australia and New Zealand	**YES**			**YES**		**YES**			**YES**
**McKinzie**	2017	Deconstruction of destruction stories: narrative, inequality, and disasters*			**YES**	**YES**	**YES**	**YES**			
**McSherry**	2015	From Deviant to Bakla, Strong to Stronger: Mainstreaming Sexual and Gender Minorities into Disaster Risk Reduction in the Philippines	**YES**	**YES**	**YES**	**YES**	**YES**	**YES**			**YES**
**Memon***	2018	Reduced Recovery of Depression in Female T Allele Carriers of TNF-RII rs1061622 at			**YES**	**YES**					

		Earlier Stage after Wenchuan Earthquake									
**Mobula***	2016	Prevalence of Hypertension among Patients Attending Mobile Medical Clinics in the Philippines after Typhoon Haiyan			**YES**	**YES**	**YES**	**YES**			
**Modena***	2017	Gender Differences in Post-Traumatic Stress		**YES**	**YES**	**YES**	**YES**	**YES**			
**Moise**	2016	Hospitalizations for Substance Abuse Disorders Before and After Hurricane Katrina: Spatial Clustering and Area-Level Predictors, New Orleans, 2004 and 2008		**YES**			**YES**	**YES**			
**Montano***	2016	Rethinking our approach to gender and disasters: Needs, responsibilities, and solutions			**YES**		**YES**	**YES**			
**Mordeno***	2016	Examining the Latent Structure of Posttraumatic Growth Between Male and Female Survivors in the Immediate Aftermath of a Flash Flood Disaster		**YES**	**YES**	**YES**	**YES**	**YES**			
**Morita***	2018	Demographic transition and factors associated with remaining in place after the 2011 Fukushima nuclear disaster and related evacuation orders		**YES**	**YES**	**YES**	**YES**	**YES**			
**Morita***	2017	Excess mortality due to indirect health effects of the 2011 triple disaster in Fukushima, Japan: a retrospective observational study		**YES**	**YES**	**YES**	**YES**	**YES**			
**Moriyama**	2018	Efficacy of group intervention involving physical activity on subjective well-being of elderly returnees after evacuation following the Great East Japan Earthquake					**YES**	**YES**			

**Moriyama***	2017	Effect of Residence in Temporary Housing After the Great East Japan Earthquake on the Physical Activity and Quality of Life of Older Survivors		**YES**	**YES**	**YES**	**YES**	**YES**			
**Mulligan***	2015	How did the Canterbury earthquakes affect physiotherapists and physiotherapy services? A qualitative study		**YES**	**YES**	**YES**	**YES**	**YES**			
**Myers***	2018	Facilitators and barriers in implementing the Minimum Initial Services Package (MISP) for reproductive health in Nepal post-earthquake	**YES**		**YES**	**YES**	**YES**	**YES**			**YES**
**N.Sattler***	2018	Natural Disasters in Indonesia: Relationships Among Posttraumatic Stress, Resource Loss, Depression, Social Support, and Posttraumatic Growth			**YES**	**YES**	**YES**	**YES**			
**Nagai***	2018	Impact of evacuation onstrends in the prevalence, treatment, and control of hypertension before and after a disaster		**YES**	**YES**	**YES**	**YES**	**YES**			
**Nagai***	2017	Lifestyle-related factors that explain disaster-induced changes in socioeconomic status and poor subjective health: A cross-sectional study from the Fukushima health management survey		**YES**			**YES**	**YES**			
**Nagashim***	2018	Changes in pulmonary function of residents in Sanriku Seacoast following the tsunami disaster from the Great East Japan Earthquake			**YES**	**YES**	**YES**	**YES**			
**Najafi***	2015	Demographic Determinants of Disaster Preparedness Behaviors			**YES**	**YES**					

		Amongst Tehran Inhabitants, Iran									
**Nakano***	2018	Associations of disaster-related and psychosocial factors with changes in smoking status after a disaster: A cross-sectional survey after the Great East Japan Earthquake		**YES**	**YES**	**YES**	**YES**	**YES**			
**Nakaya***	2016	Partners' Ongoing Treatment for Chronic Disease and the Risk of Psychological Distress after the Great East Japan Earthquake		**YES**	**YES**	**YES**	**YES**	**YES**			
**Nakhaei***	2015	Impact of disaster on women in Iran and implication for emergency nurses volunteering to provide urgent humanitarian aid relief: A qualitative study			**YES**	**YES**					
**Naseri***	2017	A primary assessment of society-based earthquake disaster mitigation in Kabul city, Afghanistan			**YES**	**YES**					
**Nazhat***	2016	Sexual and gender-based violence in natural disasters: Emerging norms			**YES**	**YES**	**YES**	**YES**			
**Ni***	2015	Factors associated with resilience of adult survivors five years after the 2008 Sichuan earthquake in China			**YES**	**YES**	**YES**	**YES**			
**Nishat***	2018	Disaster, vulnerability, and violence against women: Global findings and a research agenda for bangladesh			**YES**	**YES**	**YES**	**YES**			
**Nobakht***	2019	Risk factors of post-traumatic stress among survivors of the 2017 Iran earthquake: The importance of peritraumatic dissociation			**YES**		**YES**	**YES**			
**Nonnecke***	2017	Malasakit 1.0: A participatory online platform for crowdsourcing disaster risk			**YES**	**YES**	**YES**				

		reduction strategies in the philippines									
**Nozue***	2017	Combined associations of physical activity and dietary intake with health status among survivors of the Great East Japan Earthquake		**YES**	**YES**	**YES**	**YES**	**YES**			
**O'Connell***	2017	Emotions and beliefs after a disaster: a comparative analysis of Haiti and Indonesia		**YES**	**YES**						
**Oe***	2016	Three-year trend survey of psychological distress, post-traumatic stress, and problem drinking among residents in the evacuation zone after the Fukushima Daiichi Nuclear Power Plant accident [The Fukushima Health Management Survey]		**YES**	**YES**	**YES**	**YES**	**YES**			
**Ohira***	2016	Evacuation and risk of hypertension after the great East Japan Earthquake		**YES**		**YES**	**YES**	**YES**			
**Ohira***	2016	Effect of evacuation on body weight after the Great East Japan Earthquake		**YES**			**YES**	**YES**			
**Ohman***	2016	The (un)intended consequences of crisis communication in news media: a critical analysis		**YES**	**YES**	**YES**	**YES**	**YES**			
**Ong***	2017	Queer cosmopolitanism in the disaster zone: ‘My Grindr became the United Nations’	**YES**	**YES**	**YES**	**YES**	**YES**	**YES**			**YES**
**Onose***	2017	Sex differences in post-traumatic stress disorder in cardiovascular patients after the Great East Japan Earthquake: A report from the CHART-2 Study		**YES**	**YES**	**YES**	**YES**	**YES**			
**Orui***	2015	Delayed increase in male suicide rates in tsunami disaster-stricken areas following the great east japan earthquake: a		**YES**	**YES**	**YES**	**YES**	**YES**			

		three-year follow-up study in Miyagi Prefecture									
**Orui***	2018	Suicide Rates in Evacuation Areas After the Fukushima Daiichi Nuclear Disaster		**YES**	**YES**	**YES**	**YES**	**YES**			
**Oxfam***	2018	Gender, disaster risk reduction, and climate change adaptation: A learning companion			**YES**	**YES**	**YES**	**YES**			**YES**
**Ozoilo***	2016	Experience in the management of the mass casualty from the January 2010 Jos Crisis			**YES**	**YES**					
**Paraskevo***	2018	Post-fire attitudes and perceptions of people towards the landscape character and development in the rural Peloponnese, a case study of the traditional village of Leontari, Arcadia, Greece				**YES**	**YES**	**YES**			
**Parkinson***	2017	Investigating the Increase in Domestic Violence Post Disaster: An Australian Case Study				**YES**	**YES**	**YES**			
**Parkinson***	2015	Victoria's gender and disaster taskforce		**YES**	**YES**	**YES**	**YES**	**YES**			
**Pathak***	2017	Gendered approach towards disaster recovery: Experiences from 2011 floods in Pathumthani province, Thailand			**YES**	**YES**	**YES**	**YES**			
**Paul***	2015	Predictors of compliance with tornado warnings issued in Joplin, Missouri, in 2011			**YES**	**YES**	**YES**	**YES**			
**Paul***	2018	Fatalities caused by hydrometeorological disasters in texas			**YES**	**YES**	**YES**	**YES**			
**Payari***	2016	Sex differentials in the risk factors of post traumatic stress disorder among tsunami survivors in Tamil Nadu, India		**YES**	**YES**	**YES**	**YES**	**YES**			
**Peden**	2018	The Flood-Related Behaviour of River Users in Australia			**YES**	**YES**					
**Petrucci***	2017	The Vulnerability of People to Damaging Hydrogeological			**YES**	**YES**					

		Events in the Calabria Region (Southern Italy)									
**Piccardi***	2018	Continuous Environmental Changes May Enhance Topographic Memory Skills. Evidence From L'Aquila Earthquake-Exposed Survivors			**YES**	**YES**					
**Pledger**	2019	SF-12 indicators of health following the 22 February 2011 Christchurch earthquake					**YES**	**YES**			
**Pollack***	2016	Mental health, life functioning and risk factors among people exposed to frequent natural disasters and chronic poverty in Vietnam			**YES**	**YES**	**YES**	**YES**			
**Rakhshan***	2017	Social support for earthquake victims in East Azerbaijan, Iran			**YES**	**YES**	**YES**	**YES**			
**Rashidine***	2015	General Health Among Parents Who Lost Their Children in the Bam Earthquake			**YES**	**YES**	**YES**	**YES**			
**Reid***	2016	Differential respiratory health effects from the 2008 northern California wildfires: A spatiotemporal approach		**YES**	**YES**	**YES**	**YES**	**YES**			
**Reyes***	2017	Gender dimensions and women's vulnerability in disaster situations: A case study of flood prone areas impacting women in Malabon City, Metro Manila			**YES**	**YES**	**YES**	**YES**			
**Sado***	2018	Three-Year Follow-up After the Great East Japan Earthquake in the Incidence of Out-of-Hospital Cardiac Arrest With Cardiac Origin		**YES**	**YES**	**YES**	**YES**	**YES**			
**Sakai***	2017	Persistent prevalence of polycythemia among evacuees 4 years after the Great East Japan Earthquake: A follow-up study		**YES**			**YES**	**YES**			
**Sakai**	2015	White blood cell, neutrophil, and lymphocyte counts in					**YES**	**YES**			

		individuals in the evacuation zone designated by the government after the Fukushima Daiichi Nuclear Power Plant accident: the Fukushima Health Management Survey									
**Salvati**	2018	Gender, age and circumstances analysis of flood and landslide fatalities in Italy					**YES**	**YES**			
**Satoh***	2015	Evacuation after the Fukushima Daiichi Nuclear Power Plant Accident Is a Cause of Diabetes: Results from the Fukushima Health Management Survey			**YES**	**YES**	**YES**	**YES**			
**Satoh***	2016	Hypo-high-density Lipoprotein Cholesterolemia Caused by Evacuation after the Fukushima Daiichi Nuclear Power Plant Accident: Results from the Fukushima Health Management Survey				**YES**	**YES**	**YES**			
**Scott**	2017	Sex estimation from measurements of the calcaneus: Applications for personal identification in Thailand		**YES**	**YES**	**YES**					
**Sevimli***	2015	Health professionals of emergency service: An evaluation of disaster medicine and ethical values			**YES**	**YES**	**YES**				
**Sevilmi**	2016	Issues affecting health professionals during and after catastrophic earthquakes in Van-Turkey					**YES**	**YES**			
**Shapira***	2016	An Integrated and Interdisciplinary Model for Predicting the Risk of Injury and Death in Future Earthquakes			**YES**						
**Sharifi***	2015	The severity of cutaneous leishmaniasis before and after the earthquake in Bam, southeastern Iran			**YES**	**YES**					

**Sheikhbar***	2017	Rehabilitation of vulnerable groups in emergencies and disasters: A systematic review			**YES**		**YES**	**YES**			
**Sherman***	2017	Emergency Preparedness Safety Climate and Other Factors Associated With Mental Health Outcomes Among World Trade Center Disaster Evacuees			**YES**	**YES**					
**Shi ***	2018	Posttraumatic stress disorder symptoms in parents and adolescents after the Wenchuan earthquake: A longitudinal actor-partner interdependence model			**YES**	**YES**					
**Shieh**	2019	Rate of psychiatric disorders and associations with quality of life among community members following the Kaohsiung gas explosion: an 18-month cross-sectional follow-up study			**YES**	**YES**					
**Shooshtar***	2018	The mental health needs of women in natural disasters: A qualitative study with a preventive approach			**YES**	**YES**	**YES**				
**Shrestha***	2015	Post-traumatic Stress Disorder among Medical Personnel after Nepal earthquake, 2015			**YES**	**YES**	**YES**				
**Shreve***	2016	Conceptualizing an equitable "social framing" for economic evaluations to support gender equality in disaster risk- and environmental-management decision-making			**YES**	**YES**	**YES**	**YES**			
**Sloand***	2015	Barriers and Facilitators to Engaging Communities in Gender-Based Violence Prevention following a Natural Disaster			**YES**	**YES**	**YES**	**YES**			
**Smadi**	2016	Women's oral and dental health aspects in humanitarian					**YES**				

		missions and disasters: Jordanian experience									
**Smith***	2016	Thriving after trauma: Posttraumatic growth following the Canterbury earthquake sequence			**YES**	**YES**	**YES**	**YES**			
**Smith***	2017	In some strange way, trouble is good for people. Posttraumatic growth following the Canterbury earthquake sequence			**YES**	**YES**	**YES**	**YES**			
**Sobrabizadeh***	2016	A Qualitative Study of Violence Against Women after the Recent Disasters of Iran			**YES**	**YES**	**YES**	**YES**		**YES**	
**Sobrabizadeh***	2017	Women's Challenges and Capabilities in Disasters: A Case Report of the Twin Earthquakes of Eastern Azerbaijan, Iran			**YES**	**YES**	**YES**	**YES**			
**Sobrabizadeh***	2018	Religiosity, Gender, and Natural Disasters: A Qualitative Study of Disaster-Stricken Regions in Iran			**YES**	**YES**	**YES**	**YES**			
**Sobrabizadeh***	2016	Women and health consequences of natural disasters: Challenge or opportunity?			**YES**	**YES**	**YES**	**YES**			
**Somerfeldt***	2015	Disasters and Information Source Repertoires: Information Seeking and Information Sufficiency in Postearthquake Haiti			**YES**	**YES**	**YES**	**YES**			
**Stewart***	2017	Rural Community Disaster Preparedness and Risk Perception in Trujillo, Peru			**YES**	**YES**	**YES**				
**Stewart***	2017	Psychological Distress and Zika, Dengue and Chikungunya Symptoms Following the 2016 Earthquake in Bahia de Caraquez, Ecuado			**YES**	**YES**	**YES**	**YES**			
**Sthapit***	2015	Gendered impacts of the earthquake and responses in Nepal				**YES**	**YES**				

**Suzuki***	2015	Increased prevalence of atrial fibrillation after the Great East Japan Earthquake: Results from the Fukushima Health Management Survey				**YES**	**YES**	**YES**			
**Suzuki**	2017	Burnout among public servants after the Great East Japan Earthquake: decomposing the construct aftermath of disaster					**YES**	**YES**			
**Takahashi***	2017	Effect of evacuation on liver function after the Fukushima Daiichi Nuclear Power Plant accident: The Fukushima Health Management Surve			**YES**	**YES**	**YES**	**YES**			
**Takahashi***	2016	Weight Gain in Survivors Living in Temporary Housing in the Tsunami-Stricken Area during the Recovery Phase following the Great East Japan Earthquake and Tsunami			**YES**	**YES**					
**Takeda***	2016	Determinants of intention to leave among non-medical employees after a nuclear disaster: a cross-sectional study				**YES**	**YES**	**YES**			
**Tan***	2017	Disaster Preparedness Among University Students in Guangzhou, China: Assessment of Status and Demand for Disaster Education			**YES**	**YES**					
**Tayfur**	2018	Health results of a coup attempt: evaluation of all patients admitted to hospitals in Istanbul due to injuries sustained during the July 15, 2016 coup attempt			**YES**	**YES**					
**Tempest**	2017	Secondary stressors are associated with probable psychological morbidity after flooding: a cross-sectional analysis					**YES**	**YES**			
**Teramoto**	2015	Cross-sectional study of social support and psychological					**YES**	**YES**			

		distress among displaced earthquake survivors in Japan									
**Thapa***	2019	Gender inclusiveness in disaster risk governance for sustainable recovery of 2015 Gorkha Earthquake, Nepal			**YES**	**YES**	**YES**	**YES**			
**Thoresen***	2019	Long-term mental health and social support in victims of disaster: comparison with a general population sample			**YES**	**YES**	**YES**	**YES**			
**Trumble***	2018	Parental hormones are associated with crop loss and family sickness following catastrophic flooding in lowland Bolivia			**YES**	**YES**	**YES**	**YES**			
**Tsubota***	2018	Association between health risks and frailty in relation to the degree of housing damage among elderly survivors of the great East Japan earthquake			**YES**	**YES**	**YES**	**YES**			
**Tucker***	2017	Possible link of Interleukin-6 and Interleukin-2 with psychiatric diagnosis, ethnicity, disaster or BMI			**YES**	**YES**					
**Tyler***	2018	Gender, households, and decision-making for wildfire safety		**YES**	**YES**	**YES**					
**Uemura**	2016	Association between psychological distress and dietary intake among evacuees after the Great East Japan Earthquake in a cross-sectional study: The Fukushima Health Management Survey					**YES**	**YES**			
**Urbatsch**	2016	Judgement days: Moral attitudes in the wake of local disasters									**YES**
**Urmson***	2016	Asking for help and receiving support after a disaster			**YES**	**YES**	**YES**	**YES**			
**van Berlaer***	2018	Clinical Characteristics of the 2013 Haiyan Typhoon Victims			**YES**	**YES**					

		Presenting to the Belgian First Aid and Support Team									
**Wang***	2017	Assessment of the Public Health Risks and Impact of a Tornado in Funing, China, 23 June 2016: A Retrospective Analysis			**YES**	**YES**	**YES**				
**Wang***	2015	Assessing the underlying dimensionality of DSM-5 PTSD symptoms in Chinese adolescents surviving the 2008 Wenchuan earthquake			**YES**	**YES**					
**Weiss***	2018	Chernobyl Thyroid Cancer: 30 Years of Follow-up Overview			**YES**	**YES**					
**Wen ***	2018	Gender Differences in Psychosocial and Physical Outcomes in Haitian Amputees			**YES**	**YES**	**YES**	**YES**			
**Wisner**	2017	We've seen the future, and it's very diverse: beyond gender and disaster in West Hollywood, California	**YES**	**YES**	**YES**	**YES**	**YES**	**YES**			
**Witvorapong***	2015	Social Participation and Disaster Risk Reduction Behaviors in Tsunami Prone Areas			**YES**		**YES**	**YES**			
**Wu***	2015	A cross-sectional survey on the health status and the health-related quality of life of the elderly after flood disaster in Bazhong city, Sichuan, China		**YES**	**YES**	**YES**	**YES**	**YES**			
**Yabuki***	2015	Pain, quality of life and activity in aged evacuees living in temporary housing after the Great East Japan earthquake of 11 March 2011: a cross-sectional study in Minamisoma City, Fukushima prefecture			**YES**	**YES**	**YES**	**YES**			
**Yamashita**	2015	Segregation, exclusion and LGBT people in disaster impacted areas: experiences from the Higashinihon Dai-Shinsai (Great East-Japan Disaster)	**YES**		**YES**	**YES**	**YES**	**YES**			

**Yang***	2017	Understanding the Outcome in the Chinese Changjiang Disaster in 2015: A Retrospective Study			**YES**	**YES**	**YES**	**YES**			
**Yoshihama**	2018	Participatory Investigation of the Great East Japan Disaster: PhotoVoice from Women Affected by the Calamity					**YES**				
**Yoshihama***	2018	Violence Against Women and Children Following the 2011 Great East Japan Disaster: Making the Invisible Visible Through Research			**YES**	**YES**	**YES**	**YES**	**YES**	**YES**	
**Yoshimura***	2016	Relationships between social factors and physical activity among elderly survivors of the Great East Japan earthquake: a cross-sectional study		**YES**	**YES**	**YES**	**YES**	**YES**			
**Young***	2017	Sex, gender, and disasters: Experimental evidence on the decision to invest in resilience		**YES**	**YES**	**YES**	**YES**	**YES**			
**Yu ***	2016	Evaluation of blast injury patients from the 2015 Tianjin explosions in China			**YES**	**YES**	**YES**	**YES**			
**Yumarni ***	2018	Gender mainstreaming and sustainable development goals: A systematic literature review in post disaster reconstruction area			**YES**	**YES**	**YES**	**YES**			
**Yumarni ***	2018	Gender mainstreaming as a strategy to achieve sustainable post-disaster reconstruction			**YES**	**YES**	**YES**	**YES**			
**Yumarni**	2017	Resource capability of local governments in mainstreaming gender into disaster risk reduction: Evidence from bantul Indonesia					**YES**	**YES**			
**Zagheni***	2015	Differential mortality patterns from hydro-meteorological disasters: Evidence from cause-of-death data by age and sex		**YES**	**YES**	**YES**	**YES**	**YES**			
**Zahlawi***	2019	Psychosocial support during displacement due to a natural			**YES**		**YES**	**YES**			

		disaster: relationships with distress in a lower-middle income country									
**Zengin***	2015	Fire disaster caused by LPG tanker explosion at Lice in Diyarbakır (Turkey): July 21, 2014			**YES**	**YES**					
**Zengin***	2015	How was Felt Van Earthquake by a Neighbor University Hospital?			**YES**	**YES**					
**Zhang***	2015	Risk Factors of Posttraumatic Stress Disorder among Survivors after the 512 Wenchuan Earthquake in China			**YES**	**YES**					
**Zhang***	2015	A Cross-sectional Study on Posttraumatic Stress Disorder and General Psychiatric Morbidity Among Adult Survivors 3 Years After the Wenchuan Earthquake, China			**YES**	**YES**					
